# Black Soldier Fly Full-Fat Larvae Meal Is More Profitable Than Fish Meal and Fish Oil in Siberian Sturgeon Farming: The Effects on Aquaculture Sustainability, Economy and Fish GIT Development

**DOI:** 10.3390/ani11030604

**Published:** 2021-02-25

**Authors:** Mateusz Rawski, Jan Mazurkiewicz, Bartosz Kierończyk, Damian Józefiak

**Affiliations:** 1 Laboratory of Inland Fisheries and Aquaculture, Department of Zoology, Faculty of Veterinary Medicine and Animal Science, Poznań University of Life Sciences, Wojska Polskiego 71C, 60-625 Poznań, Poland; mateusz.rawski@up.poznan.pl; 2 Hipromine S.A., Poznańska 12F, 62-023 Robakowo, Poland; damian.jozefiak@up.poznan.pl; 3 Department of Animal Nutrition, Faculty of Veterinary Medicine and Animal Science, Poznań University of Life Sciences, Wołyńska 33, 60-637 Poznań, Poland; bartosz.kieronczyk@up.poznan.pl

**Keywords:** Siberian sturgeon, black soldier fly, aquaculture sustainability, insect meal, gastrointestinal tract development

## Abstract

**Simple Summary:**

The practical use of alternative feed materials must be both sustainable and profitable. Therefore, the aim of this study was to assess the impact of black soldier fly full-fat larvae meal (BSFL) on environmental and economic aspects as well as gastrointestinal tract (GIT) development in Siberian sturgeon nutrition. The experimentally obtained data were used for calculations of fish meal (FM) and fish oil (FO) relative usage per kg of fish gain. The economic conversion ratio and profitability per unit of fish gain were assessed also. The samples of intestinal and liver tissues were analysed in terms of villi height, width, and surface, and liver health. The study showed a high potential of BSFL usage up to its highest used incorporation that was 30% of the diet. The environmental sustainability of the diets was increased not only by the reduction in FM and FO use but also by the increase in feed conversion efficiency. Thus, even though the price of BSFL was higher than FM and FO, the profitability of fish production was improved, finding its optimum at the levels of 10% and 15% BSFL incorporation. Moreover, health beneficial effects of BSFL were observed in alterations of GIT development.

**Abstract:**

This study provides data on the environmental sustainability, economic profitability, and gastrointestinal tract development of Siberian sturgeon diets containing black soldier fly full-fat larvae meal (BSFL) for a fish meal (FM) and fish oil (FO) replacement. BSFL was used at 5%, 10%, 15%, 20%, 25%, and 30% of the diet, replacing by up to 61.3% of FM and 95.4% of FO. BSFL positively affected the feed efficiency ratio, and lowered FM and FO usage per kg of fish gain. All the BSFL diets showed a sustainable fish-in fish-out (FIFO) ratio, which was lowered by up to 75% in comparison to the control. Economic assessment per kg of fish gain showed that the most lucrative variants were variants with 10% and 15% BSFL, it finds a mode of action in improvements of the gastrointestinal tract development, including increased pyloric caeca and proximal intestine shares and enhanced villus height and area. Thus, in Siberian sturgeon, BSFL may be used not only as an FM and FO replacer but also as a functional material due to its feed utilization and beneficial health effects, which are reflected in its high sustainability and favourable economics.

## 1. Introduction

It is expected that by 2025 aquafeed production will grow by 33% compared with that in 2015 [1]. To keep its position as the leader in modern animal production development, global aquaculture must be sustainable. Therefore, reducing the environmental footprint caused by unsustainable material use in aquafeed production is a key effort [1,2]. Circular economy elements and reasonable resource use must be introduced through reducing the use of marine-derived raw materials—mainly fish meal (FM) and fish oil (FO)—and increasing the use of renewable feed ingredients. In recent decades, FM and FO shares in fish diets have been partially reduced each year; however, the global usage of marine resources by aquaculture has increased [1,2]. It occurs due to almost constant levels of FM and FO production, which cannot be raised due to marine overfishing and the increasing needs of upscaling aquaculture [3]. Currently, this sector consumes over 70% of global FM and FO resources annually, the consequence of which is the increase in FM and FO prices [2], thus a number of innovative alternative nutrient sources are under consideration [2]. In the initial phase of their development, their practical use does not find a financial balance and is limited due to high prices and low production volumes. Nevertheless, increasing attention has been given to insect biomass due to its renewable nature and low emissions, whereby production is based mainly on bioconversion of re-food and by-products [4,5]. Notably, in the last decade, insect farming has transformed from a hobbyist and basic research field estimated to be approximately 500 tonnes per year, to a continuously and rapidly upscaling branch of animal production, estimated to reach 250,000 tonnes annually by 2030 in the EU alone [6]. Consequently, the price of these alternatives will progressively decrease and availability will increase, which makes possible the use of insect-derived raw materials in farm animal nutrition. The quality of *Hermetia illucens* derived products is improved also by the number of technological procedures [7,8,9]. Moreover, EU legislation (Regulation 2017/893/EC, 2017) approves the use of insect-derived feed materials in aquaculture [10]. Currently, in the second decade of rapid development of the idea of insects used as food and feed, a number of questions have been answered in terms of their effects on animals, especially fish growth and metabolism [11,12,13,14,15,16]. In our previous publication, the effects of black soldier fly full-fat larvae meal (BSFL) on growth performance, feed physical properties, attractivity, and utilization were provided [16]. Moreover, a key improvement in the diet calculation—the application of modified nitrogen to protein conversion factor—resulted in positive effects on the abovementioned parameters [16]. However, to be relevant from a practical approach, these promising results should also be considered in terms of sustainability and profitability assessments.

It must be emphasized that in aquaculture production, environmental and economic analyses of alternative raw materials usage should not be considered as a simple idea of feed ingredients replacement. To complete this assessment and to obtain an objective point of view, fish growth performance data, including body weight gain (BWG), feed conversion ratio (FCR), feed costs, and live fish sale prices, must be included. Thus, in the current study, the empirical numbers obtained in the feed production process and in vivo experiment were used to calculate the sustainability and profitability of BSFL use in Siberian sturgeon (*Acipenser baerii*) diets.

The volume of *Acipenseridae* production is increasing worldwide; according to official reports, 250 tonnes of sturgeons are captured, while more than 100,000 tonnes are farmed annually, which is a 100% increase in comparison to the sturgeon aquaculture production from 2010 [17]. Sturgeons consume a high share of insects in their natural diets and show a high level of attraction to insect smell or taste [16,18]. Thus, the use of insect meals seems to be well justified in their nutrition; however, to date, only five reports have been published on the effects of insect meals on sturgeon nutrition [12,16,19,20,21,22]. Thus, the analysis of the physiological response of BSFL use is strongly required. This publication aims to provide the novel data important in three main areas: global aquaculture production sustainability, showing the potential of insect meals as marine independent raw materials; fish farming industry, assessing the possibility of financially profitable BSFL use; scientific description of mode of action for the results presented in the study as well as previously published data [16].

## 2. Materials and Methods

### 2.1. Ethics Statement

Studies on live animals were carried out in strict accordance with the recommendations of the National Ethics Commission (Warsaw, Poland). All members of the research staff were trained in animal care, handling, and euthanasia. At the end of the experiment, 14 animals per treatment (2 per tank) were euthanized for tissue sampling for histomorphological analysis. Fish were anaesthetized with an overdose of tricaine methanesulfonate (MS222, 300 mg L^−1^) by prolonged immersion [23]. After sedation, the animals were decapitated according to the American Veterinary Medical Association Guidelines for the Euthanasia of Animals [24]. According to Polish law and an EU directive (no 2010/63/EU), the experiments conducted in this study did not require approval (certificate given in supplementary materials).

### 2.2. Insect Meal and Diet Preparation

The BSFL was produced at HiProMine S.A., Robakowo, Poland. The larval diet, methodology of full-fat meal preparation, and nutritive values were reported by Rawski et al. [16]. The diets were calculated based on a modified nitrogen to protein conversion factor (Kp) according to Janssen et al. [25] as detailed in Rawski et al. [13]. Feeds were prepared by extrusion processing with a single-screw warm extruder (Metalchem S-60 Gliwice, Poland) at the Experimental Station of Feed Production Technology and Aquaculture in Muchocin (Poznań University of Life Sciences). The processing conditions were as follows: 90 °C cylinder temperature in the zone of increasing pressure, 110 °C cylinder temperature in the zone of high pressure, 120 °C head temperature, 52 rpm screw speed, and 3 mm nozzle diameter. In post-production, fish oil was added by vacuum coating (Rollermac BA 15 FR aut. Pomati Group S.R.L, Codogno, Italy). In total, seven diets were prepared: control (CON), containing 26.1% FM and 0% BSFL; H5, with 23.4% FM and 5% BSFL; H10, with 20.8% FM and 10% BSFL; H15, with 18.1% FM and 15% BSFL; H20, with 15.5% FM and 20% BSFL; H25, with 12.8% FM and 25% BSFL; and H30, with 10.1% FM and 30% BSFL. The diet compositions and analysed nutritive values are given in Table 1.

### 2.3. Animal Care

The growth trial was carried out using 392 Siberian sturgeon fingerlings (mean body weight 14.4 ± 3.0 g). The fingerlings were obtained from the Experimental Station of Feed Production Technology and Aquaculture in Muchocin (Poznań University of Life Sciences), and, after 7 days of acclimation, they were randomly distributed into 49 rectangular fiberglass tanks (60 dm^3^ capacity). A total of 56 fish were used per treatment, with 8 fish per tank (mean body weight 14.4 ± 2.0 g). The experiment was conducted over 50 days. The study design included seven treatments, with seven replications (tanks) per treatment with details given by Rawski et al. [16]. Feeding rate was corrected according to body weight and water temperature as described by Hung et al. [26].

### 2.4. Sustainability and Economic Assessment

All the parameters were calculated on the basis of raw data for growth performance and feed utilization parameters to provide a sufficient amount of data for statistical assessment. For each treatment, seven replications were used. Feed conversion efficiency (FCE) was used as a fish growth index of each feed, giving reversed feed conversion ratio (FCR) information about the amount of fish gain obtained with one kg of the feed, and calculated according to the formula described by Sunde et al. [27]:

FCE (g/g) = body weight gain (g)/feed intake (g)(1)

The relative usage values of marine-derived feed ingredients—fish meal (FMU) and fish oil (FOU)—were calculated according to the following formulas:

FMU (g/1 kg of fish gain) = fish meal share in the diet (g/1 kg) × (feed intake (g)/body weight gain (g))(2)

FOU (g/1 kg of fish gain) = fish oil share in the diet (g/1 kg) × (feed intake (g)/body weight gain (g))(3)

The fish-in fish-out ratio (FIFO) was calculated as the quantity of live fish from capture fisheries required for each unit of farmed fish produced [28,29,30,31,32]. This indicator was calculated as follows:

FIFO = ((level of fish meal in the diet (g/1 kg) + level of fish oil in the diet (g/1 kg)/(yield of fish meal from wild fish (g/1 kg) + yield of fish oil from wild fish (g/1 kg))) × (feed intake (g)/body weight gain (g))(4)

where the yield of fish meal from wild fish was assumed to be 225 g/1 kg of fresh fish weight and the yield of fish oil from wild fish was assumed to be 50 g/1 kg of fresh fish weight [28,29]. The value of FIFO ≤ 1 was assumed to be environmentally sustainable, i.e., not causing higher fish removal than production [30,31,32].

To determine the economic relative efficiency and benefits of the tested diet cost of feed per unit of fish gain, the economic conversion ratio (ECR) and economic profit index (EPI) were calculated using the following formulas by Stejskal et al. [29]:

ECR (€/1 kg of fish gain) = (feed intake (g)/body weight gain (g)) × cost of feed (€/1 kg)(5)

EPI (€/fish) = (body weight gain (kg) × sale price of live fish (€/1 kg)) − (body weight gain (kg) × (cost of feed (€/1 kg × (feed intake (g)/body weight gain (g))(6)

Profitability (PRO) was calculated according to the balance of fish sale price and feed costs per kg of fish gain (ECR). Other costs of fish production were not introduced to the calculation, which was performed according to the following formula:

PRO (€/1 kg of fish gain) = sale price of live fish (€/1 kg) − economic conversion ratio (€/1 kg of fish gain)(7)

The per kg costs in euros used for calculation are given in supplementary materials.

### 2.5. Somatic Indices

Somatic indices for evaluating the effect of diet on the internal organs and the fish condition were analysed according to the methodology described by Piccolo et al. [33] with some modifications for adapting the method to the Siberian sturgeon gastrointestinal tract characterization by taking under consideration pyloric caeca, as well as proximal and distal intestine separately. The indices included CF—condition factor, VSI—viscerosomatic index, HSI—hepatosomatic index, PCI—pyloric caeca index, GIT/FTL—relative gastrointestinal tract length, IL/FTL—relative intestinal length, PL/FTL—relative proximal intestine length, DL/FTL—relative distal intestine length, IL/GIT—intestine share in total gastrointestinal tract length, PL/GIT—proximal intestine share in total gastrointestinal tract length, and DL/GIT—distal intestine share in total gastrointestinal tract length. They were calculated according to the formulas presented below:

CF = (body weight (g)/fish total length (cm)^3^) × 100 (8)

VSI (%) = (viscera weight (g)/body weight (g)) × 100(9)

HSI (%) = (liver weight (g)/body weight (g)) × 100(10)

PCI (%) = (pyloric caeca weight (g)/body weight (g)) × 100(11)

GIT/FTL (%) = (gastrointestinal tract length (mm)/fish total length (mm)) × 100(12)

IL/FTL (%) = (intestines length (mm)/fish total length (mm)) × 100(13)

PL/FTL (%) = (proximal intestine length (mm)/fish total length (mm)) × 100(14)

DL/FTL (%) = (distal intestine length (mm)/fish total length (mm)) × 100(15)

IL/GIT (%) = (intestines length (mm)/gastrointestinal tract length (mm)) × 100(16)

PL/GIT (%) = (proximal intestine length (mm)/fish total length (mm)) × 100(17)

DL/GIT (%) = (distal intestine length (mm)/fish total length (mm)) × 100(18)

### 2.6. Histomorphological Examination

To measure the histopathological changes in both of the abovementioned tissues, 14 fish were sampled from each treatment (2 per tank) and subjected to 2 histological slides per each fish. The samples of the intestine were fixed by immersion in Bouin solution and stored at 4 °C for 24 h. Histological examination of the intestine was performed on samples of liver as well as proximal intestine, using the methodology described Bogucka et al. [34]. To measure the height of the villi, ten villi per fish were randomly selected from a cross-section. The length was measured from the tip of the villus to its base; the width was measured at half of its length, for all villi measurements the mean of ten measurements was calculated for statistical analysis. The surface of the villi (VS) was calculated according to the Sakamoto et al. [35] formula as follows:

VS = (2 π) × (villus width/2) × (villus height)(19)

The thickness of the muscle membrane was defined as the average of five measurements per fish. Histological examination of the liver samples was performed based on the paraffin method and haematoxylin and eosin staining.

After preparing the slides, 10 images per sample of the liver were used for the analysis of qualitative and quantitative data. A semiquantitative scoring system was used to evaluate the severity of histopathological changes in liver of Siberian sturgeons in the case of the presence of vacuoles in hepatocytes and characteristic fat vacuolizations, number of congestions, necrosis, and fibrosis. The protocol provided by Peebua et al. [36] and Elia et al. [37] with some modifications was used. The scoring system included a 5-point (0–4) scale where 0 represents no changes, 1 represents slight histopathology present in less than 25% of fields, 2 represents mild histopathology present in less than 50% of fields, 3 represents moderate histopathology present in less than 75% of fields, and 4 shows severe histopathology observed in more than 75% of fields. The examples of visualized samples are given in Figure S1.

### 2.7. Statistical Analysis

In the case of sustainability and economic assessment, the experimental unit was tank n/treatment = 7. In the case of somatic, morphological, and histomorphological analysis, 2 fish per tank were used; thus, n/treatment = 14. All obtained data were tested for normal distribution using the Kolmogorov-Smirnov test. Analysis of variance homogeneity (ANOVA) was conducted using Bartlett’s test. The significance of differences between groups was determined by Duncan’s multiple range test at a significance level of *p* ≤ 0.05. The calculations were performed using SAS 9.4 software (Cary, NC, USA). The following general model was used:

Y_i_ = μ + α_i_ + δ_ij_ (20)

where Y_i_ is the observed dependent variable; μ is the overall mean; α_i_ is the effect of insect inclusion; and δ_ij_ is the random error [16,38].

## 3. Results

### 3.1. Environmental Sustainability and Economic Profitability

The feed conversion efficiency results showed that all the BSFL-containing groups showed significantly higher fish body weight gain per kg of feed (Table 2). The highest values were observed in groups H10, H15, H20, H25, and H30. The relative FM and FO usages per kg of fish gain decreased in a higher proportion than expected according to its share in the diet. In comparison with CON diet the FMU per kg of fish gain was decreased by 19.1%, FOU by 23% in H5; in H10, by 37.0% and 46.6%; in H15, by 44.8% and 58.3%; in H20, by 53.9% and 71.3%; in H25, by 61.7% and 83.3; and in H30, by 70% and 96.5, respectively. The decrease in FMU and FOU was significantly lowered by each 5% increase in BSFL added (*p* < 0.0001). Among the treatments, only CON showed a FIFO ratio higher than 1, and each 5% BSFL dose resulted in a significant decrease in FIFO, which in H30 was 25% of the CON value (*p* < 0.0001). In the case of feed price, each additional 5% of BSFL raised the price of feed by 7.5 € cents per kg, costs of each feed were 1.72 €/kg of CON, 1.80 €/kg of H5, 1.87 €/kg of H10, 1.95 €/kg of H15, 2.02 €/kg of H20, 2.10 €/kg of H25 and 2.17 €/kg of H30. The ECR per kg of live fish was lowered in comparison to CON (1.52 €/1 kg of fish gain) by each HI treatment, with the significantly lowest values in H10 (1.30 €/1 kg of fish gain) and H15 (1.37 €/1 kg of fish gain), (*p* < 0.0001). The EPI per fish was increased (*p* < 0.0001) by all BSFL-containing treatments compared with CON (0.45 €/fish), with the highest values for H10 (0.61 €/fish), H15 (0.61 €/fish), H20 (0.63 €/fish), H25 (0.61 €/fish), and H30 (0.62 €/fish). The profit per kg of live fish was the highest (*p* < 0.0001) in the H10 (6.70 €/1 kg of fish gain) and H15 (6.70 €/1 kg of fish gain) treatments and the lowest in the CON (6.48 €/1 kg of fish gain) and H30 treatments (6.53 €/1 kg of fish gain). There were no differences between H5 (6.57 €/1 kg of fish gain), H15 (6.63 €/1 kg of fish gain), H20 (6.62 €/1 kg of fish gain), and H25 or between H25 (6.56 €/1 kg of fish gain) and H30.

### 3.2. Somatic Indices and Gastrointestinal Histomorphology

The CF compared to CON (0.31) was significantly increased (*p* = 0.0005) by H10 (0.34), H15 (0.36), H20 (0.35), H25 (0.37), and H30 (0.36) with no effect of H5 (0.33), as shown in Table 3. Viscerocomatic index (VSI) related to CON (0.16%) was increased (*p* = 0.0130) by H20 (0.22%) and H30 (0.22%). Pyloric caeca share in the body weight (PCI) compared to CON was increased (*p* = 0.0001) by all treatments in which BSFL was used. Differences were not observed (*p* = 0.1893) in the hepatosomatic index (HSI) and in the relative lengths of the total gastrointestinal tract (*p* = 0.0875), whole intestine (0.1087), proximal (*p* = 0.1900) and distal intestine (*p* = 5743). The share of the proximal intestine in total gastrointestinal tract length in comparison to CON (26.8%) was increased (*p* = 0.0079) by the H25 treatment (30.1%); in the H10 (28.1%), H15 (28.5%), H20 (28.5%), and H30 (27.5%) treatments, the PL/GIT was numerically higher than that in CON; the DL/GIT was lowered (*p* = 0.0341) by all treatments with the use of BSFL compared to CON. Histomorphological analysis (Table 4) of the intestine showed that villus height was increased in the H10 (720 µm), H20 (742 µm), H25 (802 µm), and H30 (807 µm) treatments, with the highest values in the H25 and H30 groups, compared to that in CON (*p* < 0.0001). Villi width was the highest (*p* = 0.0098) in the H20 group (100.6 µm) while it was the lowest in H30 (93.1 µm) while 97.7 µm was recorded in CON. The abovementioned results compared to CON showed increased villus surfaces in H20, H25, and H30, with the highest value in the H25 group (*p* < 0.0001). No differences in terms of muscular layer thickness were recorded (*p* = 0.1051). The histological analysis of liver tissue samples (Table 5) showed no severe or moderate changes. Congestion, fibrosis, and fat vacuoles showed slight changes, and necrosis and vacuolization showed slight to mild changes. No significant differences in terms of liver degeneration, i.e., congestion (*p* = 0.2406), necrosis (*p* = 0.5762), fibrosis (*p* = 0.7653), hepatocyte vacuolization (*p* = 0.7653), and fat vacuole presence (*p* = 0.9712), were recorded.

## 4. Discussion

The environmental impact and profitability of modern aquaculture are the most important factors considering the rapidly increasing global human population needs [3]. Thus, a wide spectrum of alternative feed ingredients is currently considered [2,4,5]. However, none of published up to date studies on Siberian sturgeon nutrition contains the analysis of sustainability or economic assessment of the experimental data. Moreover, most of them show no growth performance or feed utilization improvement caused by BSFL usage, which is probably an effect of the use of highly defatted meals [22] or lack of nitrogen to protein conversion ratio corrections [16]. The provided experimental data show the high environmental sustainability of full-fat BSFL use in Siberian sturgeon production. The main mechanism may be the increased feed conversion efficiency (FCE), which resulted in a 290 to 340 g increase in juvenile fish weight gain per 1 kg of feed compared to that in the CON treatment, when 10 to 30% BSFL was used in the feeds. This finding indicates that BSFL use may contribute to aquaculture sturgeon production efficiency increase. Due to improved feed utilization, the relative levels of FM and FO use per unit of fish gain were decreased by up to 70 and 96.5%, respectively, in H30, which is a higher level than 61.3 and 95.4% based on the feed composition change only. This suggests very strongly that the highest sustainability of BSFL use may be reached in the case of fish species in which growth performance and feed utilization parameters not only remain at the level observed in the case of FM and FO use but also are improved by insect materials. The most widely used ratio of fish production sustainability assessment—FIFO [30,31,32]—was decreased by all the experimental treatments in comparison with CON. All the results were below 1.0, which means that except for the CON treatment, none of the BSFL-containing treatments caused higher fish capture than production. For comparison, in the case of European perch diets, FIFO decreased from 3.04 in the control diet to 1.18–2.17 in the case of BSFL use [29]. It indicates that in a high FM diet without growth performance and feed utilization improvement, the balanced aquaculture fish production cannot be reached, even when 60% of BSFL/kg of feed was used due to the remaining FM at 27% of the diet [29]. The abovementioned FM share was even higher than in the present study CON treatment—26.1% of the diet. It suggests that for carnivorous and omnivorous fishes, FIFO optimization can be reached when the share of FM in the diets is in the range of 26–27% and the feed conversion ratio is close to 1.0 or lower. It must also be noted that modern diets contain progressively lower amounts of FM and FO in their composition due to their low sustainability and high costs. They are replaced by non-alternative but not marine protein and energy sources, i.e., blood meal, animal by-product meals, or plant blends [39]. Thus, in the authors’ opinion, in further experiments even control diets should be calculated in a way that assumes reaching FIFO ≤ 1.0 by the use of low FM and FO shares. However, sustainability is not enough for the application of alternative feed ingredients, and their use must meet the economic balance on the market—by price or positive effects on animal production—in terms of feed efficiency or animal health [40]. According to published reports, feed costs in Siberian sturgeon farming are 38–42.3% of total production expenses, with an average price of feed of 1.61 €/kg [41], which is comparable to the presented control feed cost (1.72 €/kg) as well as with other experimental diets [29,42]. Earlier approaches included European sea bass (*Dicentrarchus labrax*) for the entire 18-month cycle of production and mealworm (*Tenebrio molitor*) meal usage [42]. In the abovementioned study, the increase in feed price with insect meal use was clearly shown and wavered from less than 7 € cents to almost 32 € cents per kg of feed—depending on the theoretically assumed price of the meal—from 2.5 to 5.0 €/kg of mealworm meal, and an indifference price of *Tenebrio molitor* meal at the level of 1.74 €/kg with total FM substitution at 2.47€/kg when 50% of FM was replaced in a 1:1 ratio, with very similar growth performance results among the calculated treatments [42]. In the present study, the actual price at which BSFL was obtained, at the level of 3.00 €, was assumed, which resulted in a feed price increase of 7.5 € cents/kg per each 5% of BSFL inclusion. This level can be justified in the case of improvement in growth performance parameters caused by the functional character of feed material or its high nutritive value, which was observed and discussed in our previous study [16]. The economic assessment of BSFL use was performed earlier by Stejskal et al. [29], who assumed the price of BSFL at the level of 3.5 €/kg, which resulted in an almost 11 cent per kg price increase per 5% incorporation. Together with the lack of growth depression, the use of 40% BSFL in feed resulted in a similar EPI; however, feed costs per kg of produced fish (ECR) increased significantly when more than 20% BSFL was used [29]. In the present study, high sustainability was reached due to improved growth performance and feed utilization. Its explanation was partially presented in our previous publication; however, the morphological and histomorphological analyses provide additional details for mode of action. In the case of fish diets containing 10% or higher BSFL, increased body conditions were observed; however, no differences in the HSI were recorded. This may suggest a lack of effects on excessive lipid storage confirmed by lack of liver fatty degradation observed in histological analyses. The increased CI should be interpreted as a symptom of proper fish growth and development as it occurs during fish growth naturally [43]. The present study showed that BSFL inclusion in the diet affects the development of gastrointestinal tract macrostructure, including somatic indices. In comparison, Caimi et al. [16] did not observe the effect of *Hermetia illucens* defatted meal on the fish condition, HSI, and VSI up to 375 g/kg of feed. The VSI and PCI suggest the positive effects of insects in terms of gastrointestinal tract development, which may be confirmed by the increase in proximal intestine relative length. Both pyloric caeca and the proximal intestine are the main segments of the GIT responsible for enzymatic digestion as well as nutrient assimilation; thus, their development as a consequence of BSFL use may be considered a symptom of the functional role of insect-derived materials in fish nutrition. All the abovementioned results are supported by the digestibility coefficients of protein and fat, which did not decline, and improvement in fish growth performance and feed utilization [16]. The explanation of the obtained results may be observed in the simultaneous alteration of macrostructures as well as histomorphological microstructure development, represented by increased height, width, and surface of intestinal villi. These alterations may be the reason for maintaining the levels of nutrient digestibility or even probable increases in micro- and macronutrient absorption, which is expected in the intestine due to observed improvements in growth performance. This finding is confirmed by the study of Józefiak et al. [12] on Siberian sturgeon, in which full-fat BSFL successfully replaced FM, and modified gastrointestinal tract histomorphology and microbiota, which was suggested as a consequence of a diet closer to its composition in the natural environment than the fish meal-based control diet. The increase in villus height and area also suggests gut health and microbiome homeostasis, which, if unbalanced, would result in villus degeneration and erosion [20,22,35,38]. The data obtained in the presented study shows positive effects of BSFL on proximal intestine development including higher share of pyloric caeca in body weight of fish, increased proximal intestine length in relation to the gastrointestinal tract length and increase of villi height and area. This result is contrary to those of Zaranotoniello et al. [22], who observed worsening in gut histological morphometric parameters. The results of the mentioned study are partially explained by low acceptance of the diet and fish partial starvation. However, in the presented experiment the feed acceptance was improved [16] as well as its utilization by fish. Thus the feed palatability should be deeply studied in the case of BSFL use in Siberian sturgeons. The above-described morphological changes in the gastrointestinal tract may be an explanation for the constant level of digestibility coefficients presented in our previous study [13] even though the chitin level increased; it is possible that they provided a compensatory effect on its antinutritive properties due to GIT adaptation [12,44]. Another possible explanation is the effect of a high level of lauric acid in the BSFL (22 g/100 g of crude fat), which could decrease potentially pathogenic microorganism development in the gastrointestinal tract as well as act as an inflammatory inhibitor and growth performance stimulator [16,21]. Fatty liver degradation is one of the common problems in intensive sturgeon farming and may cause growth depression or even unexpectedly high mortality of fish, without earlier symptoms. It was observed earlier by Zarantoniello et al. [22] when 50% of FM was replaced by BSFL with 23% inclusion of BSFL to the diet. Thus, the condition of the liver tissue was assessed in the present study also. It showed no increase in the level of histopathological changes in liver structure due to the increasing level of BSFM in the diet. This finding aligns with that of Caimi et at. [20], who described a lack of negative effects on the liver structure with up to 37.5% black soldier fly meal inclusion. However, in the current literature, there are various effects of *Hermetia illucens* meals on liver health, described due to selection of its form, fish species, and insect meal dose [37,45,46,47]. The results of BSFL application as an alternative feed material in Siberian sturgeon diets are promising. The profitability reached a level of 10% to 15% of BSFL in the diet, while sustainability progressed with an increasing share of BSFL. The positive alteration in gastrointestinal tract macro- and microstructures resulted in an increase in the feed efficiency ratio and no negative effects on fish health, with the highest share of BSFL = 30%. Thus, further studies on BSFL use in Acipenseridae diets are highly recommended.

## 5. Conclusions

The present study showed that it is possible to reach high environmental sustainability in Siberian sturgeon farming by dietary substitution of FM and FO with BSFL. Due to improved fish growth performance and feed utilization parameters, the relative levels of FM and FO replacements may be even higher than those that would be calculated on the basis of diet composition. For the first time, in the case of a Siberian sturgeon in vivo study, it has been proven that BSFL incorporation into the fish diet may be more profitable than FM and FO application. The mode of action for growth performance and feed utilization improvements as well as high nutrient digestibility observed in the present experiment and described earlier by Rawski et al. [28] was developed with respect to gastrointestinal tract ontogenetic modulation. BSFL use improved the development of the pyloric caeca and proximal intestine as well as its microstructures due to increased villus height, width, and area, which could be the reason for the growth performance alteration. According to authors knowledge this is the first report of such a high level of sustainability and economic profitability in the case of BSFL introduction to a fish diet.

## Figures and Tables

**Table 1:** Compositions and proximate chemical analysis of experimental diets.

Treatment	CON	H5	H10	H15	H20	H25	H30
	Diet composition (g/1000 g)						
Fish meal	261	234	208	181	155	128	101
Red blood cells	100	100	100	100	100	100	100
BSFL	0	50	100	150	200	250	300
Soy protein isolate	100	100	100	100	100	100	100
Wheat gluten	150	150	150	150	150	150	150
Wheat meal	145	130	117	104	89	76	63
Maltodextrin	130	130	130	130	130	130	130
Fish oil	65	55	44	34	24	14	3
Lecithin	10	10	10	10	10	10	10
Premix ^1^	15	15	15	15	15	15	15
Vitamin premix ^2^	1	1	1	1	1	1	1
Choline chloride	2	2	2	2	2	2	2
Limestone	18	18	16	14	3	11	10
Phosphate 1-Ca	0	2	4	6	8	10	12
TiO_2_	3	3	3	3	3	3	3
Total	1000	1000	1000	1000	1000	1000	1000
	Analysed chemical feed composition (g/1000 g)						
Dry matter	938.4	937.4	934.5	934.7	934.9	936.0	935.9
Crude protein	485.5	487.7	491.4	497.9	503.5	507.4	507.9
Crude fat	99.7	102.4	101	95.5	92.1	92.3	91.0
Crude fibre	6.7	9.9	13.3	16.7	19.9	23.2	26.5
Ash	82.1	82.3	82.2	80.9	81.2	80.1	80.1
Nitrogen-free extract	264.4	255.1	246.6	243.7	238.2	233.0	230.4

^1^ Polfamix W, BASF Polska Ltd. Kutno, Poland; containing per 1 kg: vitamin A 1,000,000 IU, vitamin D 3,200,000 IU, vitamin E 1.5 g, vitamin K 0.2 g, vitamin B1 0.05 g, vitamin B2 0.4 g, vitamin B12 0.001 g, nicotinic acid 2.5 g, D-calcium pantothenate 1.0 g, choline chloride 7.5 g, folic acid 0.1 g, methionine 150.0 g, lysine 150.0 g, Fe 2.5 g, Mn 6.5 g, Cu 0.8 g, Co 0.04 g, Zn 4.0 g, and J 0.008 g. ^2^ Vitazol AD_3_EC, BIOWET Drwalew, Poland; containing per 1 kg: vitamin A 50,000 IU, vitamin D3 5000 IU, vitamin E 30.0 mg, vitamin C 100.0 mg.

**Table 2:** Sustainability and economic assessment of Siberian sturgeon production with the use of experimental diets containing increasing share of black soldier fly full-fat larvae meal.

ITEM	CON	H5	H10	H15	H20	H25	H30	SEM	*p*-Value
Environmental Sustainability									
FCE	1.13 ^c^	1.27 ^b^	1.43 ^a^	1.42 ^a^	1.47 ^a^	1.42 ^a^	1.47 ^a^	0.019	<0.001
FMU	230 ^a^	186 ^b^	145 ^c^	127 ^d^	106 ^e^	88 ^f^	69 ^g^	7.676	<0.001
FOU	57.3 ^a^	43.6 ^b^	30.6 ^c^	23.9 ^d^	16.4 ^e^	9.58 ^f^	2.03 ^g^	2.605	<0.001
FIFO	1.04 ^a^	0.83 ^b^	0.64 ^c^	0.55 ^d^	0.44 ^e^	0.35 ^f^	0.26 ^g^	0.037	<0.001
Economic Profitability									
FC	1.72	1.80	1.87	1.95	2.02	2.10	2.17	-	-
ECR	1.52 ^a^	1.43 ^bc^	1.30 ^d^	1.37 ^cd^	1.38 ^c^	1.44 ^bc^	1.47 ^ab^	0.013	<0.001
EPI	0.45 ^c^	0.53 ^b^	0.61 ^a^	0.61 ^a^	0.63 ^a^	0.61 ^a^	0.62 ^a^	0.010	<0.001
PRO	6.48 ^d^	6.57 ^bc^	6.70 ^a^	6.63 ^ab^	6.62 ^b^	6.56 ^bc^	6.53 ^cd^	0.013	<0.001

FCE—feed conversion efficiency (g/g), FMU—relative fish meal use (g/1 kg of fish gain), FOU—relative fish oil use (g/1 kg of fish gain), FIFO—fish-in fish-out ratio, FC—feed cost (€/kg), ECR—economic conversion ratio (€/1 kg of fish gain), EPI—economic profitability index (€/fish), PRO—profitability (€/1 kg of fish gain); CON—control feed, H5—experimental feed with 5% black soldier fly full-fat larvae meal, H10—experimental feed with 10% black soldier fly full-fat larvae meal, H15—experimental feed with 15% black soldier fly full-fat larvae meal, H20—experimental feed with 20% black soldier fly full-fat larvae meal, H25—experimental feed with 25% black soldier fly full-fat larvae meal, H30—experimental feed with 30% black soldier fly full-fat larvae meal. Different letters (a,b,c,d,e,f,g) indicate differences between treatments (*p* ≤ 0.05).

**Table 3:** Somatic indices in Siberian sturgeons fed experimental diets containing increasing shares of black soldier fly full-fat larvae meal.

ITEM	CON	H5	H10	H15	H20	H25	H30	SEM	*p*-Value
CF	0.31 ^d^	0.33 ^cd^	0.34 ^bc^	0.36 ^abc^	0.35 ^abc^	0.37 ^a^	0.36 ^ab^	0.004	0.0005
VSI (%)	0.16 ^b^	0.17 ^b^	0.20 ^ab^	0.19 ^ab^	0.22 ^a^	0.20 ^ab^	0.22 ^a^	0.005	0.0130
HSI (%)	3.26	3.73	3.75	3.64	3.35	3.33	3.42	0.064	0.1893
PCI (%)	0.40 ^b^	0.54 ^a^	0.60 ^a^	0.61 ^a^	0.55 ^a^	0.66 ^a^	0.61 ^a^	0.015	0.0001
GIT/FTL (%)	65.8	68.5	68.2	71.16	69.1	70.1	70.0	0.488	0.0875
IL/FTL (%)	35.2	35.4	36.3	37.3	36.2	38.2	36.5	0.300	0.1087
PL/FTL (%)	17.6	18.3	19.1	19.7	19.7	21.1	19.3	0.230	0.1900
DL/FTL (%)	17.6	17.0	17.2	17.6	16.5	17.1	17.2	0.163	0.5743
IL/GIT (%)	53.6	51.6	53.2	52.6	52.4	54.6	52.2	0.320	0.2144
PL/GIT (%)	26.8 ^b^	26.7 ^b^	28.1 ^b^	28.5 ^ab^	28.5 ^ab^	30.1 ^a^	27.5 ^b^	0.263	0.0079
DL/GIT (%)	26.8 ^a^	24.8 ^b^	25.2 ^b^	24.9 ^b^	23.9 ^b^	24.5 ^b^	24.7 ^b^	0.231	0.0341

CF—condition factor, VSI—viscerosomatic index, HSI—hepatosomatic index, PCI—pyloric caeca index, GIT/FTL—relative gastrointestinal tract length, IL/FTL—relative intestinal length, PL/FTL—relative proximal intestine length, DL/FTL—relative distal intestine length, IL/GIT—intestinal share in total gastrointestinal tract length, PL/GIT—proximal intestine share in total gastrointestinal tract length, DL/GIT—distal intestine share in total gastrointestinal tract length; Experimental design as described in the Table 2. Different letters (a,b,c) indicate differences between treatments (*p* ≤ 0.05).

**Table 4:** Intestinal histomorphology assessment in Siberian sturgeons fed experimental diets containing increasing shares of black soldier fly full-fat larvae meal.

ITEM	CON	H5	H10	H15	H20	H25	H30	SEM	*p*-Value
VH (µm)	690 ^c^	707 ^cb^	720 ^b^	715 ^cb^	742 ^b^	802 ^a^	807 ^a^	9.136	<0.0001
VW (µm)	97.7 ^abc^	94.1 ^bc^	98.8 ^ab^	95.6 ^abc^	100.6 ^a^	98.9 ^ab^	93.1 ^bc^	1.162	0.0098
VS (µm^2^)	214,296 ^c^	208,396 ^c^	221,771 ^bc^	215,272 ^c^	232,865 ^ab^	249,766 ^a^	235,655 ^ab^	260.5	<0.0001
MLT (µm)	127.7	117.3	122.7	117.1	127.1	123.0	130.7	11.64	0.1051

VH—villi height, VW—villi width, VS—villi surface, MLT—muscular layer thickness; length; Experimental design as described in the Table 2. Different letters (a,b,c) indicate differences between treatments (*p* ≤ 0.05).

**Table 5:** Histological assessment of liver samples in Siberian sturgeon fed experimental diets containing increasing shares of black soldier fly full-fat larvae meal.

ITEM	CON	H5	H10	H15	H20	H25	H30	SEM	*p*-Value
Congestion	0.21	0.57	0.50	0.21	0.36	0.14	0.14	0.010	0.2406
Necrosis	0.86	0.64	0.86	1.29	1.21	0.93	0.64	0.021	0.5762
Fibrosis	0.36	0.43	0.71	0.64	0.43	0.43	0.50	0.016	0.7653
Hepatocyte vacuolization	1.36	1.50	1.57	1.21	1.14	1.00	1.5	0.026	0.7653
Fat vacuolization	0.5	0.57	0.57	0.36	0.36	0.43	0.57	0.017	0.7912

Assessment with use of a 5-point (0–4) scale, where 0 represents no changes, 1 represents slight histopathology present in less than 25% of fields, 2 represents mild histopathology present in less than 50% of fields, 3 represents moderate histopathology present in less than 75% of fields, and 4 shows severe histopathology observed in more than 75% of fields; length; Experimental design as described in the Table 2.

## Data Availability

All the data discussed in the manuscript are present within the text and tables; detailed data for fish growth performance results and feed physical properties are published in Rawski, M., Mazurkiewicz, J., Kierończyk, B., & Józefiak, D. (2020). Black Soldier Fly Full-Fat Larvae Meal as an Alternative to Fish Meal and Fish Oil in Siberian Sturgeon Nutrition: The Effects on Physical Properties of the Feed, Animal Growth Performance, and Feed Acceptance and Utilization. Animals, 10(11), 2119. DOI: 10.3390/ani10112119.

## Supplementary Materials

The following are available online at https://www.mdpi.com/2076-2615/11/3/604/s1, Figure S1: Examples of histopathological changes observed in Siberian sturgeon liver.

## References

[1] Salin K.R., Arun V.V., Nair C.M., Tidwell J.H., Fisal I.H., Visvanathan C., Bopathy R. (2018). Sustainable aquafeed. Sustainable Aquaculture.

[2] Gasco L., Gai F., Maricchiolo G., Genovese L., Ragonese S., Bottari T., Caruso G., Gasco L., Gai F., Maricchiolo G., Genovese L., Ragonese S., Bottari T., Caruso G. (2018). Fishmeal alternative protein sources for aquaculture feeds. Feeds for the Aquaculture Sector: Current Situation and Alternative Sources.

[3] FAO (2018). The State of World Fisheries and Aquaculture 2018-Meeting the Sustainable Development Goals.

[4] Nogales-Mérida S., Gobbi P., Józefiak D., Mazurkiewicz J., Dudek K., Rawski M., Kierończyk B., Józefiak A. (2019). Insect meals in fish nutrition. Rev. Aquac..

[5] van Huis A., Oonincx D. (2017). The environmental sustainability of insects as food and feed. A review. Agron. Sustain. Dev..

[6] IPIFF Edible Insects on the European Market. https://ipiff.org/wp-content/uploads/2020/06/10-06-2020-IPIFF-edible-insects-market-factsheet.pdf.

[7] Huang C., Feng W., Xiong J., Wang T., Wang W., Wang C., Yang F. (2019). Impact of drying method on the nutritional value of the edible insect protein from black soldier fly (*Hermetia illucens* L.) larvae: Amino acid composition, nutritional value evaluation, in vitro digestibility, and thermal properties. Eur. Food Res. Technol..

[8] Wang T., Shen Q., Feng W., Wang C., Yang F. (2020). Aqueous ethyl acetate as a novel solvent for the degreasing of black soldier fly (*Hermetia illucens* L.) larvae: Degreasing rate, nutritional value evaluation of the degreased meal, and thermal properties. J. Sci. Food Agric..

[9] Zhu D., Huang X., Tu F., Wang C., Yang F. (2020). Preparation, antioxidant activity evaluation, and identification of antioxidant peptide from black soldier fly (*Hermetia illucens* L.) larvae. J. Food Biochem..

[10] Official Journal of the European Union Commission Regulation (EU) 2017/893. https://eur-lex.europa.eu/legal-content/EN/TXT/PDF/?uri=CELEX:32017R0893&from=EN.

[11] Józefiak A., Nogales-Merida S., Mikołajczak Z., Rawski M., Kierończyk B., Mazurkiewicz J. (2019). The utilization of full-fat insect meal in rainbow trout (*Oncorhynchus mykiss*) nutrition: The effects on growth performance, intestinal microbiota and gastrointestinal tract histomorphology. Ann. Anim. Sci..

[12] Józefiak A., Nogales-Mérida S., Rawski M., Kierończyk B., Mazurkiewicz J. (2019). Effects of insect diets on the gastrointestinal tract health and growth performance of Siberian sturgeon (*Acipenser baerii* Brandt, 1869). BMC Vet. Res..

[13] Mikołajczak Z., Rawski M., Mazurkiewicz J., Kierończyk B., Józefiak D. (2020). The effect of hydrolyzed insect meals in sea trout fingerling (*Salmo trutta* m. *trutta*) diets on growth performance, microbiota and biochemical blood parameters. Animals.

[14] Weththasinghe P., Hansen J.Ø., Nøkland D., Lagos L., Rawski M., Øverland M. (2021). Full-fat black soldier fly larvae (*Hermetia illucens*) meal and paste in extruded diets for Atlantic salmon (*Salmo salar*): Effect on physical pellet quality, nutrient digestibility, nutrient utilization and growth performances. Aquaculture.

[15] Gasco L., Józefiak A., Henry M. (2020). Beyond the protein concept: Health aspects of using edible insects on animals. J. Insects Food Feed.

[16] Rawski M., Mazurkiewicz J., Kierończyk B., Józefiak D. (2020). Black soldier fly full-fat larvae meal as an alternative to fish meal and fish oil in Siberian sturgeon nutrition: The effects on physical properties of the feed, animal growth performance, and feed acceptance and utilization. Animals.

[17] EUMOFA (European Market Observatory for Fisheries and Aquaculture Products) (2019). The Caviar Market. Production, Trade and Consumption in and Outside the EU.

[18] Kasumyan A., Williot P., Nonnotte G., Vizziano-Cantonnet D., Chebanov M. (2018). Olfaction and gustation in *Acipenseridae*, with special references to the Siberian sturgeon. The Siberian sturgeon (Acipenser baerii, Brandt, 1869) Volume 1–Biology.

[19] Caimi C., Renna M., Lussiana C., Bonaldo A., Gariglio M., Meneguz M., Dabbou S., Schiavone A., Gai F., Elia A.C. (2020). First insights on black soldier fly (*Hermetia illucens* L.) larvae meal dietary administration in Siberian sturgeon (*Acipenser baerii* Brandt) juveniles. Aquaculture.

[20] Caimi C., Gasco L., Biasato I., Malfatto V., Varello K., Prearo M., Pastorino P., Bona M.C., Francese D.R., Schiavone A. (2020). Could dietary black soldier fly meal inclusion affect the liver and intestinal histological traits and the oxidative stress biomarkers of Siberian sturgeon (*Acipenser baerii*) juveniles?. Animals.

[21] Gasco L., Finke M., Huis A. (2018). Can diets containing insects promote animal health?. J. Insects Food Feed.

[22] Zarantoniello M., Randazzo B., Nozzi V., Truzzi C., Giorgini E., Cardinaletti G., Freddi L., Ratti S., Girolametti F., Osimani A. (2020). Physiological responses of Siberian sturgeon (Acipenser baerii) juveniles fed on full-fat insect-based diet in an aquaponic system. Sci. Rep..

[23] Topic Popovic N., Strunjak-Perovic I., Coz-Rakovac R., Barisic J., Jadan M., Persin Berakovic A., Sauerborn Klobucar R. (2012). Tricaine methane-sulfonate (MS-222) application in fish anaesthesia. J. Appl. Ichthyol..

[24] Leary S., Underwood W., Anthony R., Cartner S. (2013). AVMA Guidelines for the Euthanasia of Animals: 2013 Edition.

[25] Janssen R.H., Vincken J.P., van den Broek L.A., Fogliano V., Lakemond C.M. (2017). Nitrogen-to-protein conversion factors for three edible insects: *Tenebrio molitor*, *Alphitobius diaperinus*, and *Hermetia illucens*. J. Agric. Food Chem..

[26] Hung S.S.O., Lutes P.B., Shqueir A.A., Conte F.S. (1993). Effect of feeding rate and water temperature on growth of juvenile white sturgeon (*Acipenser transmontanus*). Aquaculture.

[27] Sunde J., Eiane S.A., Rustad A., Jensen H.B., Opstvedt J., Nygård E., Venturini G., Rungruangsak-Torrissen K. (2004). Effect of fish feed processing conditions on digestive protease activities, free amino acid pools, feed conversion efficiency and growth in Atlantic salmon (*Salmo salar* L.). Aquac. Nutr..

[28] Crampton V.O., Nanton D.A., Ruohonen K., Skjervold P.O., El-Mowafi A. (2010). Demonstration of salmon farming as a net producer of fish protein and oil. Aquac. Nutr..

[29] Stejskal V., Tran H.Q., Prokesova M., Gebauer T., Giang P.T., Gai F., Gasco L. (2020). Partially defatted *Hermetia illucens* larva meal in diet of eurasian perch (*Perca fluviatilis*) juveniles. Animals.

[30] Tacon A.J.G., Metian M. (2008). Global overview of the use of fish meal and fish oil in industrially compounded aquafeeds: Trends and Future Prospects. Aquaculture.

[31] Naylor R.L., Hardy R.W., Bureau D.P., Chiu A., Elliott M., Farrell P.A., Forster I., Gatlin D.M., Goldburg R.J., Hua K. (2009). Feeding aquaculture in an era of finite resources. Proc. Natl. Acad. Sci. USA.

[32] Jackson A. (2009). Fish In-Fish Out, Ratios Explained. Aquac. Eur..

[33] Piccolo G., Iaconisi V., Marono S., Gasco L., Loponte R., Nizza S., Bovera F., Parisi G. (2017). Effect of *Tenebrio molitor* larvae meal on growth performance, *in vivo* nutrients digestibility, somatic and marketable indexes of gilthead sea bream (*Sparus aurata*). Anim. Feed Sci. Technol..

[34] Bogucka J., Dankowiakowska A., Elminowska-Wenda G., Sobolewska A., Szczerba A., Bednarczyk M. (2016). Effects of prebiotics and synbiotics delivered in ovo on broiler small intestine histomorphology during the first days after hatching. Folia Biol..

[35] Sakamoto K., Hirose H., Onizuka A., Hayashi M., Futamura N., Kawamura Y., Ezaki T. (2000). Quantitative study of changes in intestinal morphology and mucus gel on total parenteral nutrition in rats. J. Surg. Res..

[36] Peebua P., Kruatrachue M., Pokethitiyook P., Kosiyachinda P. (2006). Histological effects of contaminated sediments in Mae Klong River tributaries, Thailand, on Nile tilapia, Oreochromis niloticus. Sci. Asia.

[37] Elia A.C., Capucchio M.T., Caldaroni B., Magara G., Dörr A.J.M., Biasato I., Biasibetti E., Righetti M., Pastorino P., Prearo M. (2018). Influence of *Hermetia illucens* meal dietary inclusion on the histological traits, gut mucin composition and the oxidative stress biomarkers in rainbow trout (*Oncorhynchus mykiss*). Aquaculture.

[38] Rawski M., Kierończyk B., Długosz J., Świątkiewicz S., Józefiak D. (2016). Dietary probiotics affect gastrointestinal microbiota, histological structure and shell mineralization in turtles. PLoS ONE.

[39] Hua K., Cobcroft J.M., Cole A., Condon K., Jerry D.R., Mangott A., Praeger C., Vucko M.J., Zeng C., Zenger K. (2019). The future of aquatic protein: Implications for protein sources in aquaculture diets. One Earth.

[40] Luna M., Llorente I., Cobo Á. (2019). Integration of environmental sustainability and product quality criteria in the decision-making process for feeding strategies in seabream aquaculture companies. J. Clean. Prod..

[41] Veveris A., Hazners J., Benga E. Perspective development of new species in Latvian aquaculture. Proceedings of the 2016 International Conference Economic Science for Rural Development, LLU ESAF.

[42] Arru B., Furesi R., Gasco L., Madau F.A., Pulina P. (2019). The introduction of insect meal into fish diet: The first economic analysis on European sea bass farming. Sustainability.

[43] Craig J.M., Thomas M.V., Nichols S.J. (2005). Length–weight relationship and a relative condition factor equation for lake sturgeon (Acipenser fulvescens) from the St Clair River system (Michigan, USA). J. Appl. Ichthyol..

[44] Henry M., Gasco L., Piccolo G., Fountoulaki E. (2015). Review on the use of insects in the diet of farmed fish: Past and future. Anim. Feed Sci. Technol..

[45] Belghit I., Liland N.S., Waagbø R., Biancarosa I., Pelusio N., Li Y., Krogdahl Å., Lock E.J. (2018). Potential of insect-based diets for Atlantic salmon (*Salmo salar*). Aquaculture.

[46] Zarantoniello M., Randazzo B., Gioacchini G., Truzzi C., Giorgini E., Riolo P., Gioia G., Bertolucci C., Osimani A., Cardinaletti G. (2020). Zebrafish (*Danio rerio*) physiological and behavioural responses to insect-based diets: A multidisciplinary approach. Sci. Rep..

[47] Li S., Ji H., Zhang B., Tian J., Zhou J., Yu H. (2016). Influence of black soldier fly (*Hermetia illucens*) larvae oil on growth performance, body composition, tissue fatty acid composition and lipid deposition in juvenile Jian carp (*Cyprinus carpio* var. Jian). Aquaculture.

