# Supplementary material for: Black Soldier Fly Full-Fat Larvae Meal Is More Profitable Than Fish Meal and Fish Oil in Siberian Sturgeon Farming: The Effects on Aquaculture Sustainability, Economy and Fish GIT Development
Source: Animals (Basel). 2021 Feb 25;11(3):604. doi: 10.3390/ani11030604 (PMC7996161; doi:10.3390/ani11030604)
Supplement: Supplementary file 1 [file animals-11-00604-s001.pdf]

animals

Supplementary materials

Black Soldier Fly Full-Fat Larvae Meal is More Profitable than Fish Meal and Fish Oil in Siberian Sturgeon Farming: The Effects on Aquaculture Sustainability, Economy and Fish GIT Development

Mateusz Rawski [1], Jan Mazurkiewicz [1,2*], Bartosz Kierończyk [3] and Damian Józefiak[2,3]

[1] Laboratory of Inland Fisheries and Aquaculture, Department of Zoology, Faculty of Veterinary Medicine and Animal Science, Poznań University of Life Sciences, Wojska Polskiego 71C, 60-625, Poznań, Poland; mateusz.rawski@up.poznan.pl
[2] Hipromine S.A., Poznańska 12F, 62-023, Robakowo, Poland; damian.jozefiak@up.poznan.pl
[3] Department of Animal Nutrition, Faculty of Veterinary Medicine and Animal Science, Poznań University of Life Sciences, Wołyńska 33, 60-637, Poznań, Poland; bartosz.kieronczyk@up.poznan.pl
* Correspondence: jan.mazurkiewicz@up.poznan.pl +48 61 848 77 21

Costs used for economic assessment calculations:

The prices of feed materials from commercial local retail sales points (sales area: Western Poland) from which materials were obtained in September 2019. The feed costs were calculated excluding labour and energy used for manufacturing. The material costs per kilogram were as follows:

Fish meal = € 1.85
Black soldier fly larvae meal = € 3.00
Red blood cells = € 2.22
Soy protein isolate = € 1.29
Wheat gluten = € 1.80
Wheat meal = € 0.78
Maltodextrin = € 1.33
Fish oil = € 1.62
Lecithin = € 10.22
Premix = € 5.33
Vitamin premix = € 5,33
Choline chloride = € 10.00
Limestone = € 0.16
Phosphate 1-Ca = € 0.78
TiO_2 = € 6.67

The Siberian sturgeon sale price was calculated according to offers of 10 Polish sturgeon farmers at € 8.00/1 kg.

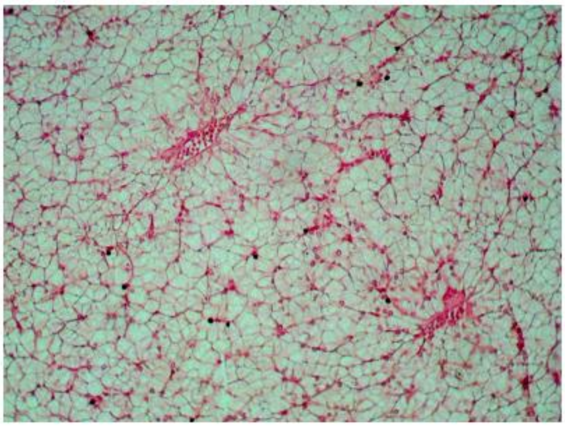

a. Proper structure of the liver, H+E staining, 100x magnification

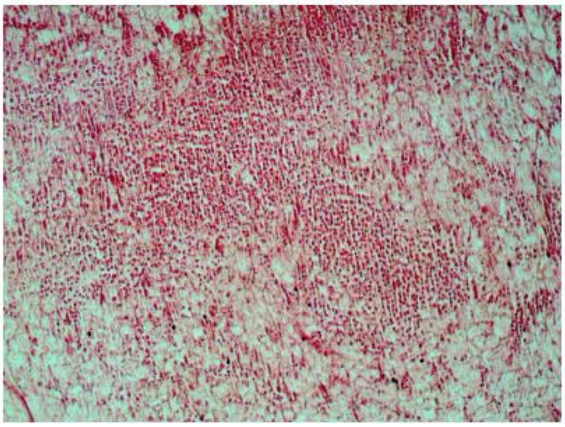

b. Congestion, H+E staining, 200x magnification

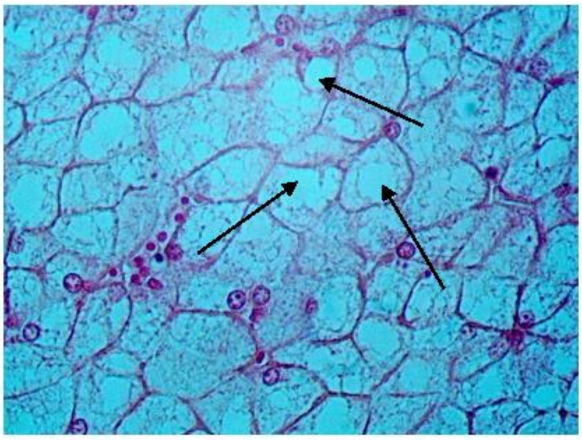

c. Vacuoles in hepatocytes (black arrows), H+E staining, 400x magnification

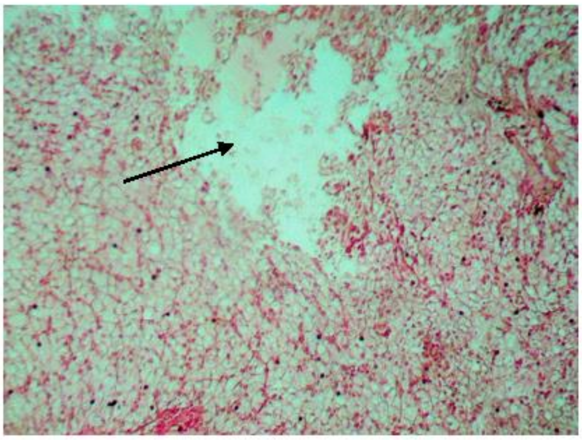

d. Necrosis (black arrow), H+E staining, 100x magnification

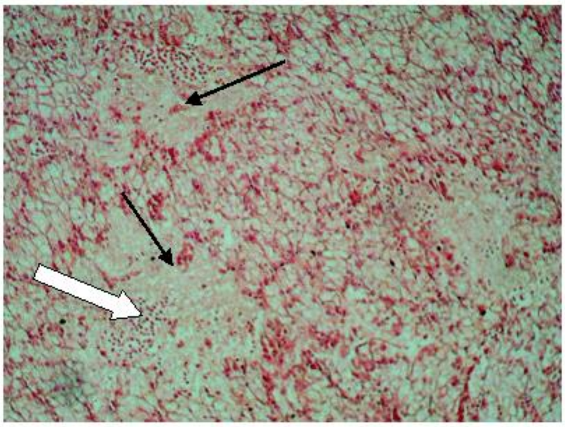

e. Necrosis with phagocytosis (white arrow), fibrosis (black arrows), H+E staining, 100x magnification

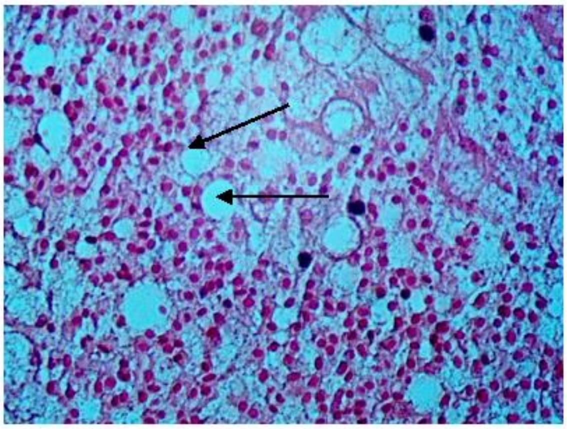

f. Fat vacuoles (black arrows), H+E staining, 200x magnification

Figure S1. Examples of histopathological changes observed in Siberian sturgeon liver.

LOKALNA KOMISJA ETYCZNA
do Spraw Doświadczeń na Zwierzętach
Uniwersytet Przyrodniczy w Poznaniu
60-637 Poznań, ul. Wołyńska 35
tel. 61 8487138, tel. 61 8466085

Paweł A Kolodziejski
Head of
The Local Ethical Commission
for Investigations on Animals in Poznań,
Poznań University of Life Sciences
Wołyńska 35
60-637 Poznań, Poland

Poznań, 2021-02-18

I certify, that according to the Act on the Protection of Animals Used for Scientific or Educational Purposes in Poland adopted on 15th January 2015 and according to earlier regulations, the studies described in the project of Mateusz Rawski, Jan Mazurkiewicz, Bartosz Kierończyk, Damian Józefiak from (Department of of Zoology, Division of Inland Fisheries and Aquaculture, Poznań University of Life Sciences, Poznań, Poland) entitled "*Black Soldier Fly Full-Fat Larvae Meal is More Profitable than Fish Meal and Fish Oil in Siberian Sturgeon Farming: The Effects on Aquaculture Sustainability, Economy and Fish GIT Development*" do not require permission of the Local Ethical Commission for Investigations on Animals.

PRZEWODNICZĄCY
Lokalnej Komisji Etycznej
do Spraw Doświadczeń na Zwierzętach
Paweł Kołodziejski
dr Paweł Kołodziejski
